# De-escalation antibiotic therapy alleviates organ injury through modulation of NETs formation during sepsis

**DOI:** 10.1038/s41420-021-00745-0

**Published:** 2021-11-10

**Authors:** Zehua Duan, Tian Xie, Chengnan Chu, Fang Chen, Xinyu Wang, Jieshou Li, Weiwei Ding

**Affiliations:** 1 Division of Trauma and Surgical Intensive Care Unit, Affiliated Jinling Hospital, Medical School of Nanjing University, Nanjing, 210002 Jiangsu Province PR China; 2 Department of Hepatobiliary and Pancreatic Surgery, Union Shenzhen Hospital, Huazhong University of Science and Technology, Shenzhen, 518000 Guangdong Province PR China; 3 School of Medicine, Southeast University, Nanjing, 210002 Jiangsu Province PR China; 4 Division of Trauma and Surgical Intensive Care Unit, the First School of Clinical Medicine, Southern Medical University, Nanjing, Jiangsu Province PR China

**Keywords:** Immune cell death, Infection

## Abstract

Empiric broad-spectrum antimicrobials therapy is suggested to be started immediately for sepsis patients. Empiric antimicrobial therapy should be narrowed once pathogen identification and sensitivities are established. However, the detailed mechanisms of de-escalation strategy are still unclear. Here we hypothesized neutrophil extracellular traps (NETs) played an essential role and de-escalation strategy might alleviate organs injury through regulation of NETs formation in sepsis. We evaluated the effect of imipenem and ceftriaxone on NETs formation in vitro and examined the role of reactive oxygen species (ROS). Next, we designed de-escalation and escalation strategy in cecum ligation and puncture (CLP) models. Organ injury, inflammatory cytokines, NETs levels were compared and evaluated. In CLP models, de-escalation therapy resulted in an increased serum MPO-DNA level during the early stage and decreased MPO-DNA level during late stage, which exerted the reverse effects in escalation therapy. Inflammatory response and organ injury exacerbated when eliminated NETs with DNAse I during the early stage of sepsis (*p* < 0.01). Histopathological analysis showed decreased injury in lung, liver, and intestine in de-escalation therapy compared with escalation therapy (*p* < 0.01). De-escalation therapy results in the highest 6-day survival rate compared with the control group (*p* < 0.01), however, no significant difference was found between de-escalation and escalation group (*p* = 0.051). The in vitro study showed that the imipenem could promote, while the ceftriaxone could inhibit the formation of NETs in PMA-activated PMNs through a ROS-dependent manner. We firstly demonstrate that de-escalation, not escalation, therapy reduces organ injury, decreases inflammatory response by promoting NETs formation in the early stage, and inhibiting NETs formation in the late stage of sepsis.

## Introduction

Sepsis remains a distressing public health care problem and ranks among the top ten causes of death worldwide. It is characterized by organ dysfunction and caused by a dysregulated host response to infection [1]. Currently, researches show that immunoparalysis is more than the overwhelming pro-inflammatory response that endangers critically ill patients [2]. The mechanisms of sepsis-induced immunoparalysis remain unclear, but functional defects of leukocytes, excessive expression of inhibitory receptors, and dysregulated production of cytokines may play an important role in the immune dysfunction in sepsis [2].

Empiric broad-spectrum therapy with one or more intravenous antimicrobials should be started immediately for patients presenting with sepsis [3]. Broad-spectrum antimicrobial therapy should be narrowed when pathogen identification and sensitivities have been established or discontinued if a decision is made that the patient does not have an infection, which was termed as de-escalation therapy. The link between early administration of antibiotics for suspected infection and antibiotic stewardship remains an essential aspect of high-quality sepsis management [4]. Although both de-escalation and escalation antibiotic therapy kill bacteria during sepsis [5], it is unclear why de-escalation, not escalation, therapy reduces mortality, and the detailed mechanism by which these changes in the sequence of antibiotic drug administration or the changes in the drugs themselves affect clinical outcomes are also unknown.

Neutrophil extracellular traps (NETs) are a new antimicrobial function of neutrophils; NETs are web-like structures accompanied by many proteins, histones, and DNA [6]. The formation of NETs is a double-edged sword in sepsis; NETs trap pathogens in the early stage, while cause NETs-associated injuries, such as coagulation, thrombotic disorders, and organ injury, in the later stage [7–9]. The balance between NETs formation and clearance plays a crucial role in sepsis. Treatments that target the clearance of NETs in the late stage of sepsis, such as DNase I and Cl-amidine, have been confirmed to ameliorate the severity of sepsis [10, 11]. In addition, NETs have been shown to link innate and adaptive immune responses by regulating the activation of apoptosis in CD4 + and CD8 + T cells [12]. In lipopolysaccharide-induced activation of monocytes, NETs can downregulate the maturation of monocyte-derived dendritic cells, thus reducing the production of cytokines (TNF-α, IL-6, IL-12, and IL-23) [13]. These may be potential mechanisms of the role of NETs in sepsis.

Previous studies have shown that some antibiotics themselves may exert immunomodulatory effects on phagocytes, cytokines, immunoglobulins, and cellular immunity [14, 15]. Although specific immunomodulatory therapy targeting inflammatory cytokines has been confirmed in sepsis models, clinical trials on the blockade of TNF, IL-1, and other cytokines failed [16]. Recent studies focused on the optimization of immunomodulatory effects of NETs formation during sepsis, enhancement of NETs formation to trap and eradicate all bacteria in the early stage, and the attenuation of excessive NETs formation to prevent NETs-associated injury in the later stage [17]. In this study, we hypothesized that antibiotics might manifest both antimicrobial and immunomodulatory functions in the treatment of sepsis and that de-escalation antibiotic therapy might alleviate organ damage and inflammatory responses through the modulation of NETs formation in the different stages of sepsis.

## Results

### De-escalation antibiotic therapy alleviates organ injury and inflammation in the CLP model

In order to compare the therapeutic effects of different antibiotic therapy methods on sepsis, mice were divided into four groups: the de-escalation group, the escalation group, the sham group, and the control group. In the control group, mice received CLP operations without any antibiotic therapy. In the de-escalation group, the imipenem was used in the first 3 days and the ceftriaxone was used in the last 3 days after CLP operation. In the escalation group, ceftriaxone was used in the first 3 days and the imipenem was used in the last 3 days.

**Fig. 1:**
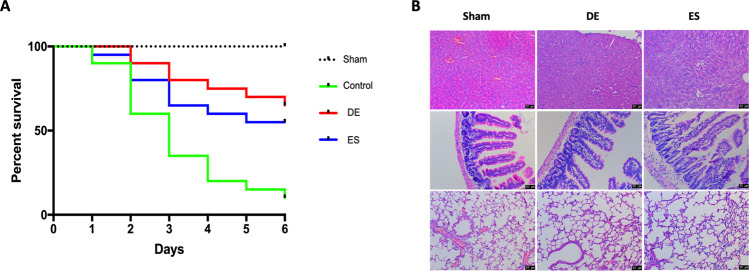
Survival rates and morphologic changes in liver, intestine, and lung tissues, in late sepsis stage (day #6). **A** Survival rates of CLP sepsis mice treated with the DE or the ES method comparing with the control group at day 6 after CLP operations. Although there was no significant difference between the DE and the ES group (*p* = 0.051), the DE has the greatest survival benefits compared with other groups. **B** Mice in sepsis groups showed more leukocyte infiltration and hydropic degeneration in liver tissues, in addition, severe inflammatory cells infiltration and microvilli damages were found in intestine tissues, and more inflammatory cells infiltration were found in lung tissues. The DE treatment presented alleviated pathological changes than the ES treatment. DE de-escalation. ES escalation. *N* = 10 per experimental group and *N* = 5 in sham group.

Most of the mice in the control group dead in 6 days after the operation. Compared with the control, antibiotic administration was more effective, but de-escalation therapy in the CLP mice did not lead to significantly higher survival than escalation therapy (*p* = 0.051, Fig. 1A). To elucidate the relationship between antibiotic therapy methods and organ injury in the late stage of sepsis, we performed histological staining of samples from the lung, intestine, and liver. The damage in the sepsis groups was significantly worse than that in the sham group, and compared with escalation therapy, de-escalation therapy reduced intra-alveolar hemorrhage, interstitial edema, and acute inflammatory cell infiltration. The lung injury score in the de-escalation group was lower than that in the escalation group (Fig. 1B).

**Fig. 2:**
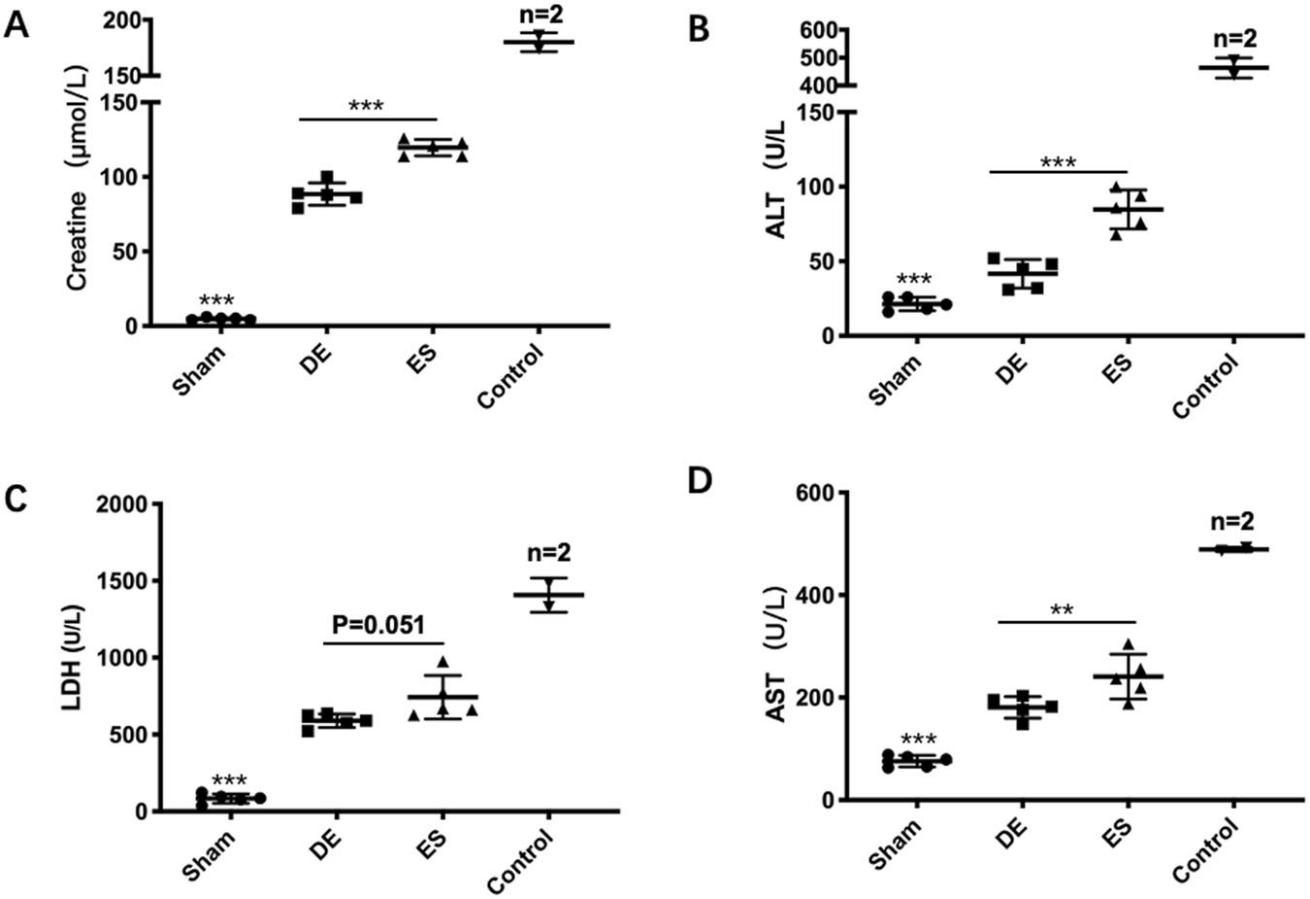
De-escalation strategy alleviates organ injuries in the late sepsis stage. **A**–**D** The serum levels of the AST, creatine, ALT, and LDH were measured with an ELISA kit in all groups (*n* = 17). The levels of blood chemistries were lower in the DE group than those in the ES group (*p* < 0.01). However, those were extremely higher in the control group though there were only 2 of 10 left on day 6. DE de-escalation. ES escalation.

**Fig. 3:**
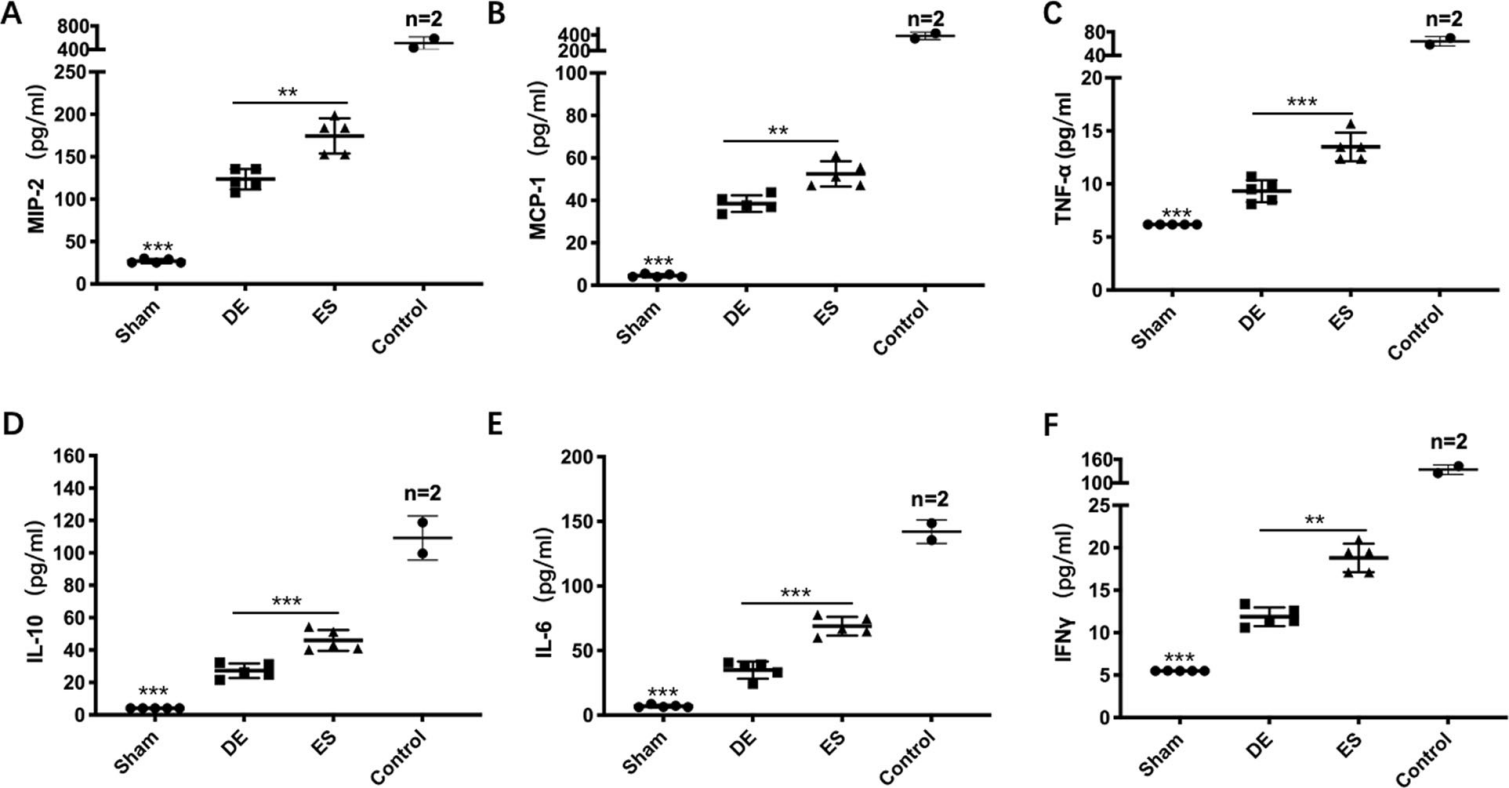
De-escalation strategy allays inflammatory response in the late sepsis stage. **A**–**F**. The serum levels of MIP-2, MCP-1, TNF-α, IL-10, IL-6, and IFN-γ were measured using Luminex^®^ xMAP (*n* = 17). The inflammatory cytokines levels were lower in the DE group than that in the ES group (*p* < 0.01), which was coincident with that in the early sepsis stage. And in the control group (*n* = 5), those biomarkers were extremely higher than those in antibiotic groups. DE de-escalation. ES escalation.

Next, we measured the AST, ALT, LDH, and serum creatinine levels to evaluate organ injury in the late stage of sepsis. There were varying degrees of damage in the sepsis group compared with the sham group, and all the serum biochemical values were lower in the de-escalation group than that in the escalation group (Fig. 2A–D). The levels of the cytokines IL-6, IL-10, IFN-γ, MIP-2, and MCP-1 were consistent with the biochemical values in the two groups (Fig. 3A–F).

These findings proved that de-escalation antibiotic therapy alleviates organ injury and inflammation in sepsis.

### De-escalation antibiotic therapy modulated the formation of NETs during sepsis

**Fig. 4:**
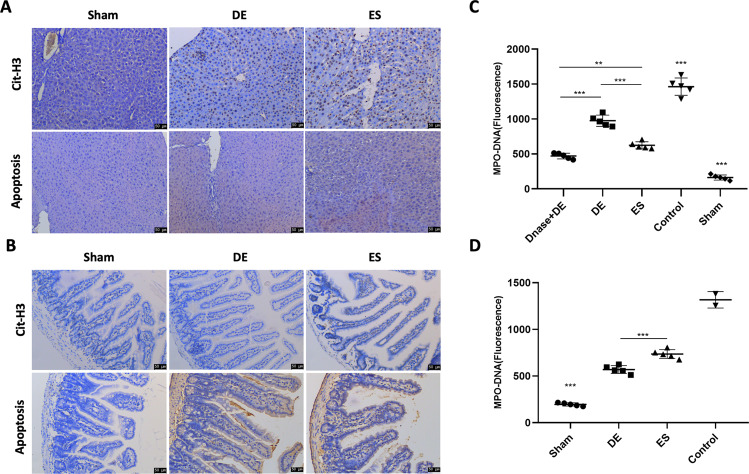
De-escalation antibiotic therapy modulated the formation of NETs during sepsis. **A**, **B** The liver and intestine tissues collected in each group were treated with the IHC examination. Severer liver and intestine injuries were correlative to more positive citH3 and apoptotic cells in these tissues. The de-escalation group had milder liver and intestine injuries and lower NETs than the escalation group. **C** At the early stage of sepsis (Day 3), blood was drawn from different groups (*n* = 25), and serum MPO-DNA levels were determined with a mouse MPO ELISA kit. DE group showed higher serum MPO-DNA levels than the ES group, and both were lower than the control group (*p* < 0.001). Higher MPO-DNA levels were diminished with the administration of DNase I (*p* < 0.001). **D** At the late stage of sepsis (Day 6), serum MPO-DNA levels were determined the same way (*n* = 20). The ES group showed higher levels of serum MPO-DNA than the DE group (*p* < 0.01), and both were higher than the sham group (*p* < 0.001). DE de-escalation. ES escalation.

In the liver and intestine samples, we compared the results of the histological and immunohistochemical analyses of citH3 and apoptosis. The injury, injury scores, and apoptosis levels were closely associated with citH3 in the liver and intestine samples of those groups. The de-escalation group had milder liver and intestine injuries and lower NETs than the escalation group (Fig. 4A, B).

To evaluate the function of NETs during different sequential antibiotic therapy, we determined the serum level of MPO-DNA, which is a biomarker of NETs, in the CLP septic model. Mice were divided into two groups: the early stage of sepsis (the timepoint was set to 72 h after the initial CLP) and the late stage of sepsis (the timepoint was set to 6 days after the initial CLP). In the early stage, the mice were divided into four groups: the de-escalation group (imipenem for 3 days), the escalation group (ceftriaxone for 3 days), the de-escalation + DNase I group (used to disrupt the NETs), and the control group. The serum MPO-DNA levels in the control mice were much higher than those in the antibiotic-treated mice (*P* < 0.01), and the MPO-DNA levels in the de-escalation group were higher than those in the escalation group (Fig. 4C). When DNase I administration was added, MPO-DNA in the de-escalation group decreased to levels lower than those in the escalation group. Next, we determined the MPO-DNA levels in the late stage of sepsis. Three groups were established: the de-escalation group, the escalation group, and the sham group. In the late stage, the serum levels of MPO-DNA in the de-escalation group were lower than those in the escalation group with the adaption of de-escalation therapy (Fig. 4D).

All the data suggested that de-escalation antibiotic therapy increased NETs formation during the early stage of sepsis and decreased NETs formation during the late stage of sepsis, which was consistent with the in vitro results described above.

### NETs play a vital role in the therapeutic effect of the de-escalation antibiotic therapy

**Fig. 5:**
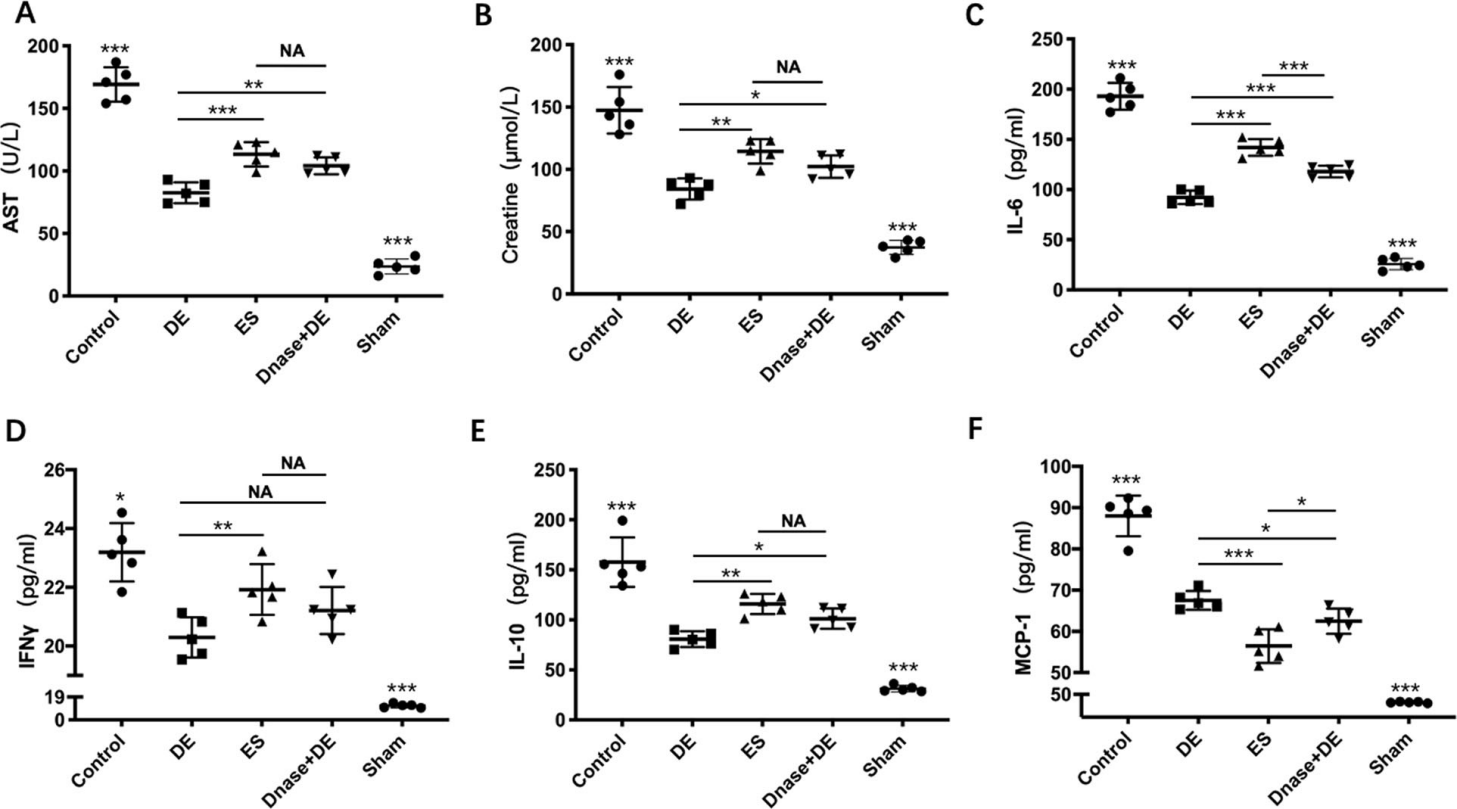
De-escalation strategy caused less organ injuries and inflammatory response attenuated by DNase I in the early sepsis stage. **A**, **B** The serum AST and Creatine were determined by ELISA (*n* = 25). Reduced levels of AST and Creatine were shown in DE compared with the ES group (*p* < 0.01), however, this effect was reversed by NETs scavenger, DNase I (*p* < 0.001). **C**–**F** The serum levels of IL-6, IFN-γ, IL-10, and MCP-1 were determined using Luminex^®^ xMAP (*n* = 25). The inflammatory cytokines levels were lower in the DE group than that in the ES group (*p* < 0.01), except for the level of MCP-1 (*p* < 0.001). When DNase I was administrated, all were elevated compared with the DE group (*p* < 0.05). DE de-escalation. ES escalation.

In the early stage of sepsis, both the levels of AST and serum creatinine were significantly lower in the de-escalation group (imipenem for 3 days) than in the escalation group (ceftriaxone for 3 days) and the control group (Fig. 5A, B). However, when DNase I was administered to the de-escalation group for 3 days, the levels of AST and serum creatinine were significantly elevated in this group. Next, we measured the inflammatory response. Consistent with the levels of AST and serum creatinine, the levels of serum IL-6, IL-10, and IFN-γ were lower in the de-escalation group than in the other groups (Fig. 5C–E). Interestingly, the level of MCP-1 was lower in the escalation group. Then, we used DNase I in the de-escalation group. The levels of all the cytokines were elevated, and the level of MCP-1 was decreased (Fig. 5F).

These findings suggested that NETs exert a protective role in the early stage of sepsis. All the data suggested that de-escalation antibiotic therapy positively regulates immune function by increasing NETs formation during the early stage of sepsis and negatively regulates immune function by decreasing NETs formation to minimize the associated overwhelming and excessive tissue injury during the late stage of sepsis.

### β-lactam antibiotics modulate the formation of NETs in PMA-activated PMNs through a ROS-dependent manner

**Fig. 6:**
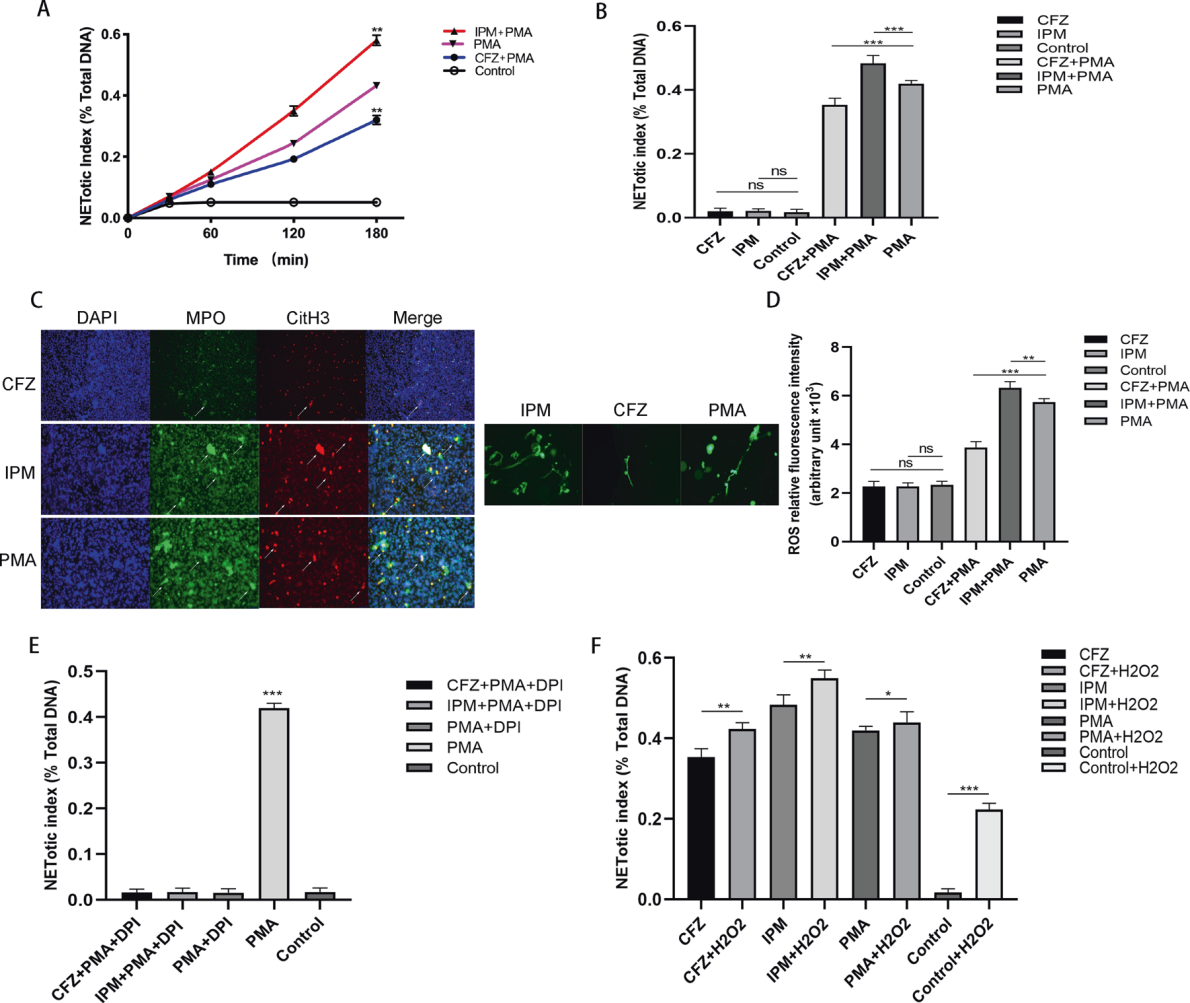
The effects of antibiotics on ROS generation and NETs formation. Human peripheral neutrophils were preincubated with antibiotics or PBS for 2 h and stimulated to form NETs with or without PMA (100 nM) for 3 h. **A** Kinetics of NETs releasing in PMA-stimulated neutrophils was recorded (*n* = 3). **B** NETs release in activated (with PMA) or resting (antibiotics alone) neutrophils was measured using Sytox Green fluorescence plate reader assay at 3 h and expression as the ratio of the percentage of total DNA (*n* = 3). Both antibiotics group showed effects on the NETs formation only in activated neutrophils. **C** To further confirm the existence of NETs, neutrophils were preincubated with antibiotics or PBS for 2 h and stimulated to form NETs with PMA (100 nM) for 3 h. DNA (blue) stained with DAPI, MPO (green) stained with anti-MPO antibody, and citH3 (red) stained with anti-citH3 were counterstained in neutrophils for immunofluorescence. DNA stained with Sytox Green alone in neutrophils for immunofluorescence to show the morphology of NETs. **D** Neutrophils were preincubated with β-Lactams (2 mM) for 2 h and then treated with or without PMA. Intracellular ROS were measured with DHR123 at 45 min (*n* = 3). **E** Neutrophils pre-cultivated with DPI, an NADPH oxidase inhibitor, were incubated with β-Lactams for 2 h and then activated with PMA. The ROS production was measured using DHR123 fluorescence plate reader assay at a time of 45 min (*n* = 3). **F** To confirm the role of ROS, exogenous H_2_ O_2_ (30 μM) was added after 2-h incubation with β-Lactams. NETosis was measured using Sytox Green fluorescence plate reader assay at the time of 3 h and expression as relative fluorescence unit (*n* = 3). Images are representative of three independent experiments. Bars were shown in the figure; data were analyzed by Student’s *t*-test. *P < 0.05, ** *P* < 0.01, ***P < 0.001, ns no significance. DE de-escalation. ES escalation.

To determine the potential effect of β-lactam antibiotics on NETs formation, we preincubated purified peripheral blood neutrophils with imipenem and ceftriaxone for 2 h. Then, the neutrophils were activated with phorbol 12-myristate 13-acetate (PMA) for 3 h. The NETotic index curves are shown in Fig. 6A. Unlike the activated neutrophils, the neutrophils (without PMA) treated with antibiotics alone did not form NETs (Fig. 6B). To confirm the existence of NETs, both fluorescence and immunofluorescence images were obtained to determine the morphology and composition of the NETs (Fig. 6C). All the data suggested that imipenem and ceftriaxone have opposite effects on the formation of NETs in activated neutrophils. The imipenem could promote, while the ceftriaxone could inhibit the formation of NETs in PMA-activated PMNs.

NADPH oxidase (NOX) is a crucial enzyme in the process of NETs formation; during the classic process of NETs formation, which proceeds in a ROS-dependent manner, NOX may modulate the generation of ROS. β-Lactams were proven to influence the activity of NOX. Thereafter, we detected the activity of NOX to explore the mechanism by which β-lactam antibiotics participate in NETs formation. DHR123, an indicator of ROS, was used to measure the generation of ROS. In both the resting and activated (activated by PMA) neutrophils, the levels of ROS were increased in the imipenem group and decreased in the ceftriaxone group compared with the control group (Fig. 6D). Then, we used diphenyleneiodonium chloride (DPI), a kind of NOX inhibitor, to explore the relationship between ROS and NETs formation in response to β-lactam antibiotics (Fig. 6E). Consistent with the results described above, DPI inhibited the formation of NETs in all the groups. Once the exogenous ROS H_2_O_2_ was added, the formation of NETs increased in all the groups (Fig. 6F), which indicated that ROS are involved in β-lactam antibiotic-induced NETs formation.

All these data showed that β-lactam antibiotics modulate the formation of NETs in activated neutrophils through a ROS-dependent manner.

## Discussion

Past clinical experience has shown that patients with sepsis benefit from de-escalation antibiotic therapy, but the mechanism has not been explored in depth. This study is the first time to elaborate the mechanism of de-escalation antibiotic therapy from animal experiments. Our results show that different β-Lactams have different effects on NETs formation both ex vivo and in vivo, and we demonstrated that de-escalation antibiotic therapy, including the early use of NETs-promoting antibiotics and the late use of NETs-inhibiting antibiotics, could fully exploit the immunomodulatory effects of NETs formation during the different stages of sepsis. More importantly, these results underline the important role of NETs directed initial empirical antibiotic therapy in the infection of polymicrobial sepsis in vivo.

Similar to previous studies, our data emphasize the protective effects of NETs in the early stage and the deleterious role of NETs in the late stage of sepsis [17, 18]. Neutrophils serve as the first line of defense in the innate immune system against bacterial pathogens. The release of intracellular contents, which is called neutrophil extracellular traps, is critical for killing bacteria in infection and sepsis [6]. Current studies have shown that NETs and their elements can cause organ injury, coagulation disorders, autoimmune diseases, and thromboembolism [19]. Although NETs are a double-edged sword in sepsis, few studies have focused on their antibacterial function in the early stage of sepsis, during which NETs can trap pathogens and prevent bacterial dissemination [17, 20, 21]. Our data show clearing NETs with DNase I will increase organ damage, these results prove that NETs formation plays an important role in organ protection during the early stage of sepsis.

We demonstrated, for the first time, that in addition to macrolide and quinolone drugs, β-lactam antibiotics can also modulate the formation of NETs in vivo and in vitro. Previous studies have proven that some antibiotics may exert immunomodulatory functions, and with the discovery of NETs, more antibiotics have been found to affect the immune system [22–24]. However, no studies have shown the immunomodulatory functions of β-lactam antibiotics, and some studies have shown a lack of immunomodulatory function of β-lactam antibiotics. This study has proven that different subgroups of β-lactam antibiotics have distinct effects on NETs formation and that imipenem and ceftriaxone regulate NETs in a ROS-dependent manner. ROS generation is a well-recognized step in NETs formation, and many studies have shown that NETs-associated diseases may be alleviated by the usage of ROS scavengers or inhibitors [25]. The β-lactam antibiotic-induced NETs regulation observed in this study could be inhibited by NOX inhibitors or promoted by exogenous ROS, which confirmed the involvement of ROS.

De-escalation therapy has been shown to be associated with reduced mortality in patients with severe sepsis and septic shock and has been shown to be effective and safe; however, there is no adequate, direct evidence or high-quality RCT studies to prove these claims, and the necessary studies to elucidate the mechanism of de-escalation therapy have not been conducted [26]. Although there is no consensus when the ridgeline of the early and late stage of sepsis is, it is acknowledged that the pathogen is confirmed 24–72 h after sepsis diagnoses. Thus, in our study, we defined that 3 days were the early stage according to the unknown pathogen period and discover whether the initial empiric antibiotic act differently. We confirmed that although it cannot improve the 6-day mortality of CLP mice in our study, de-escalation therapy can reduce organ injury and inflammatory responses, which is consistent with observations in human patients. According to the guidelines of sepsis treatment, antibiotics were administered 1 h after the CLP procedure and de-escalated. Escalation therapy was used under the guidance of empirical antibiotic usage in our study. We showed better reductions in the AST, ALT, serum creatine, and cytokine levels (IL-6, IL-10, TNF-α, IFN-γ, MIP-2, and MCP-1) in both the early and late stages of sepsis.

Based on the recovery of the function of β-lactam antibiotics in the regulation of NETs, we then explored the mechanism of de-escalation therapy. In our study, we found that the combination of different antibiotics affects the serum levels of MPO-DNA, which is a biomarker of NETs. With the sequential treatment with NETs-promoting and NETs-inhibiting antibiotics, NETs formation could be regulated, ultimately maximizing the function of NETs and reducing their damage. In the experiment with imipenem-ceftriaxone de-escalation therapy, NETs formation was higher in the early stage and lower in the late stage, which was accompanied by milder organ injury and inflammatory responses. In addition, the administration of DNase I with de-escalation therapy reduced the formation of NETs, but organ injury and inflammatory responses were exacerbated. To confirm the role of NETs, we conducted histological and immunohistochemical staining, and with greater infiltration of citH3, which is a credible NETs biomarker, increased injury to organs and enhanced apoptosis of tissue cells were observed. It seems, therefore, that in addition to the useful antibacterial function of antibiotics, the function of de-escalation therapy in the regulation of NETs is more important in sepsis; this regulatory role enhances the antibacterial function of innate immunity and prevents further NETs-associated damage in sepsis. The current guidelines suggested the completion of the sepsis bundle immediately, which included antimicrobials, nutritional therapy, and organ disfunction support [5]. In our study, there was no difference in survival between the de-escalation and the escalation group due to the sample size. On the other hand, the antibiotic treatment alone is not the whole content of the sepsis bundle strategy. However, the inflammatory cytokines levels in the de-escalation group were significantly decreased combined with the improvement of organ function. These results show the benefits of de-escalation antibiotic therapy strategies in sepsis.

However, there are some limitations. A polymicrobial sepsis model was used and we did not examine the bacterial loads and differentiate bacterial infiltration. It is important to detect the antibacterial spectrum, which takes about 24–72 h to confirm in clinical practice. The initial administration of different antibiotics may shift bacterial colonization with a certain resistance to NETs-dependent killing and influence the real outcome. Besides, bacterial colonization changes may also affect homeostasis in the intestine which contributes to the injury of the intestine. Those speculative assumptions need to be addressed in future studies. Lastly, we assessed the 6-day mortality in the escalation group which was shorter compared with the real clinical setting. Extended observation time may be expected.

## Conclusion

In this study, we firstly demonstrated that de-escalation therapy played a novel immunomodulatory effect during sepsis, which was dependent on the NETs formation. The early use of NETs-promoting antibiotics (maximizing the capacity of capturing bacteria) in the early stage, followed by NETs-inhibiting antibiotics in the late stage (preventing NETs-associated organ injury), maybe a reasonable treatment algorithm for the treatment of sepsis. Our study may shed lights on immunotherapy treatment in sepsis that involves the detection of NETs and usher the treatment of sepsis into the era of precision medicine and individualized therapy.

## Methods

### Definitions

#### De-escalation therapy

De-escalation therapy was defined as either a switch to a narrower spectrum agent or the reduction in the number of antibiotics or the early arrest of antibiotic treatment [5]. The guidelines recommend that using broad-spectrum antibiotics when the pathogen is not identified [4], otherwise using narrow-spectrum antibiotics. In our study, antibiotic de-escalation therapy is defined as the use of broad-spectrum antibiotic (imipenem) for the first 3 days and then switch to narrow-spectrum antibiotic (ceftriaxone) according to previous research [5, 7], because the bacterial culture results usually take 48–72 h to obtain.

#### Escalation therapy

Escalation therapy is the opposite of de-escalation therapy. In our study, we defined escalation therapy as the use of narrow-spectrum antibiotics (ceftriaxone) in the first three days of sepsis, followed by a broad-spectrum antibiotic (imipenem).

### Neutrophil isolation

Peripheral blood collected from healthy volunteers was layered on Polymorphprep (Axis-Shield, Norway) and centrifuged for 30 min at 500×*g*. The lower interphase having granulocytes was collected and washed with PBS. For purification, twice lysis of red blood cells was done using the red blood cell lysis buffer (Beyotime, Shanghai, China) followed by two washes with cold PBS. Isolated PMNs were suspended in RPMI 1640 (Gibco, NY, USA) medium containing 1% HEPES (Gibco, NY, USA). The protocol was approved by the institutional ethics committee at Jinling Hospital (No. 2019-NJGKJ-047).

### Animals

Adult male C57BL/6 J mice weighing between 25 and 30 g were purchased from the Experimental Animal Center, Jinling Hospital, Nanjing, China. All the experimental procedures in our study were reviewed and approved by the Institutional Animal Care and Use Committee of Jinling Hospital. All the mice received food and water ad libitum.

### Sepsis models

CLP sepsis models were established as described in ref. [7, 10]. Briefly, the mice were anesthetized using 3.5% isoflurane in 100% oxygen. A midline laparotomy was used, and the cecum was ligated with a 4–0 silk suture located at 50% between the ileocecal junction and the distal end of the cecum. A through-and-through puncture was made using a 21-G needle. A small amount of the cecal contents were extruded to ensure the patency of the hole before returning the cecum to the abdominal cavity. The peritoneum was then closed. After surgery, the mice were resuscitated with 0.9% sterile saline (500 ml SC). The mice received buprenorphine (0.05 mg/kg SC) as an analgesic. The sham animals underwent the same procedure except for the ligation and puncture of the cecum. The control animals did not undergo surgery and were euthanized alongside the CLP animals.

### Study design

Mice were randomly divided into two independent experiment studies, early sepsis stage experiment and late sepsis stage experiment. The early sepsis stage was defined as 3 days after CLP procedure and late sepsis stage was defined as 6 days or more in terms of antibiotics recommendation in sepsis and study design [4, 5, 7].

In the early sepsis experiment, mice were randomly divided into five groups, as followed: DE for de-escalation group, ES for escalation group, DNase + DE for DNase I + de-escalation group, Sham group, and Control group. In DE, ES, and DNase + DE group, imipenem (Merck & Co., New Jersey, USA) (ip.,25 mg/kg, twice a day), ceftriaxone (Pfizer, New York, USA) (ip.,75 mg/kg, twice a day), and imipenem (ip.,25 mg/kg, twice a day) + DNase I (Thermo Fisher, Waltham, MA, USA)(ip.,5 mg/kg, once a day) were used during all 3 days after CLP procedure and no antibiotics or DNase I were used in the control group. There was no CLP procedure for the sham group. Animal were euthanized on day 4 morning. Blood and organs were collected for further analysis.

In the late sepsis experiment, mice were randomly divided into four groups as DE for de-escalation group, ES for escalation group, Sham group, and Control group. In the first 3 days, the antibiotic usage was the same as the early sepsis experiment. On day 4 after the CLP procedure, ceftriaxone (ip.,75 mg/kg, twice a day) was used in the DE group while imipenem (ip.,25 mg/kg, twice a day) was used in the ES group. Namely, imipenem-initial and ceftriaxone-initial strategy were evaluated. Animals were euthanized on day 7 morning. Blood and organs were collected for further analysis.

### Cytokines and MPO-DNA quantification

Blood was collected into EDTAK2 anticoagulant (10% v/v). After centrifugation of whole blood at 1000 G for 5 min at 4 °C, plasma was collected, and the cell pellet was removed. NETs-associated MPO was captured on coated 96-well plates from the mouse MPO ELISA kit (Abcam, Cambridge, UK, Roche, Basel, Switzerland). After washes, the incubation buffer containing a peroxidase-labeled antieDNA monoclonal antibody (dilution 1:10) were added to each well. Then, the peroxidase substrate (ABTS) was added after several washes. Last, absorbance at a wavelength of 405 nm was measured. Cytokines: Interleukin (IL)-6, IL-10, interferon-γ (IFN-γ), tumor necrosis factor-alpha (TNF-α), macrophage inflammatory protein 2 (MIP-2), and monocyte chemoattractant protein-1(MCP-1) were measured using Luminex^®^ xMAP according to the manufacturer’s instructions. A standard curve was used to calculate the concentrations of cytokines.

### Determination of biochemical indexes

On day 3 and day 6 after the last administration, blood samples were taken from the peripheral blood of the mice and centrifuged to obtain the serum. The alanine transferase (ALT), aspartic acid transferase (AST), creatinine (Cr), and lactate dehydrogenase (LDH) activities in the serum were detected according to the kit (Amyjet Scientific, Wuhan, China) instructions.

### HE staining

The lung, liver, and intestine tissues of the mice were fixed with 10% neutral formalin, embedded with paraffin, sliced, stained with hematoxylin-eosin (HE), and observed under a light microscope. A semi-quantitative analysis was carried out in a blinded fashion by an experienced pathologist who was unaware of the groups to quantify the results, defined as: 0 (normal), + (mild), ++ (moderate), and +++ (severe) histological changes. The tissues and parameters assessed included the lungs (thickening of the septum, edema, congestion, and intestinal leukocyte infiltration); the liver (enlarged sinusoids, increased volume of endothelial cells, luminal leukocyte infiltration, hydropic degeneration, Kupffer cell hypertrophy, and hyperplasia) and the intestine (edema of mucosal villi, infiltration of necrotic epithelial and inflammatory cells, injury of intestinal glands, blood, and lymph vessels expanded).

### Immunohistochemistry

CitH3 has been identified as an important biomarker of NETs. Thus, immunohistochemistry analysis was performed to determine the expression levels of citH3. Immunohistochemistry was performed using standard protocols. Briefly, paraffin sections were deparaffinized and rehydrated, washed three times with PBS (PH7.4). Sections were placed in 3% hydrogen peroxide solution was incubated for 10 min. After washing with PBS, 4% goat serum was added dropwise to block, at room temperature for 30 min. Add a sufficient amount of primary antibody (CitH3, Abcam, Cambridge, MA, USA) to the section and place it in a humidified box, and incubate at room temperature for 2 h. After washing three times with PBS, add secondary antibody (goat anti-rabbit IgG, Abcam, Cambridge, MA, USA) and incubate for 30 min. Finally, stain with DAB solution and observe under the microscope, the positive signal is brown.

### Detection of cell apoptosis

Apoptosis of liver and intestine cells was detected by terminal deoxynucleotidyl. Transferase dUTP nick-end labeling assay (TUNEL) with the In-Situ Cell Death Detection kit (Roche, Basel, Switzerland) according to the kit instructions. Briefly, the cells on coverslips were washed with PBS and fixed with 4% paraformaldehyde for 15 min at room temperature. After washing, the cells were incubated in permeabilization solution (0.1% Triton X-100 in 0.1% sodium citrate, freshly prepared) for 2 min on ice. The cells were then incubated with the TUNEL reaction mixture in a humidified chamber at 37 °C for 1 h. Subsequently, the cells were briefly rinsed with PBS and counterstained with DAPI for 5 min in order to visualize the nuclei.

### NETs quantification and ROS detection

We followed the methods of Chu et al. 2020 and Xie et al. 2021 [20, 27]. In brief, the cells were seeded at 5.2 × 10^4^ cells per well in a 96-well plate in the culture media and were preincubated with antibiotics for 2 h at 37 °C in 5% CO2. Then, PMA (100 nM) was added in the presence of 5 μM SYTOX Green cell-impermeable nucleic acid stain (Life Technologies). The fluorescence intensity (RFU) was measured using a SpectraMax i3x fluorescence microplate reader (Molecular Devices LLC) at specific time intervals for up to 200 min after the activation of the cells. For the inhibitory assays, an extra 1-h preincubation with DPI was needed. The NETotic index was used to calculate the index of NETs formation and is expressed as the RFU ratio of each group. Some cells were lysed with 0.5% Triton X-100 at each time point, and this sample represented 100% DNA release.

In order to quantify reactive oxygen species (ROS) generation, isolated PMNs were suspended in RPMI 1640 medium containing 1% HEPES and pretreated with Dihydrorhodamine 123 (DHR123, 1 ummol/L, Beyotime, Shanghai, China) for 30 min. Then washing PMNs three times with PBS after centrifugation. The same number of PMNs were then resuspended with PBS and transferred into a black 96-well microplate. Fluorescence intensity was quantified every 10 min after being treated with PMA, which using SpectraMax i3x fluorescence microplate reader (Molecular Devices LLC) at an excitation wavelength of 488 nm and an emission wavelength of 530 nm.

### NETs visualization

NETs visualization was performed according to previous protocols [27]. The neutrophils used for the plate reader assay were fixed with 4% PFA in PBS buffer for 30 min and permeabilized with 0.1% Triton X-100 for 30 min. Then, blocking was performed with 10% normal goat serum for 30 min at RT. The cells were stained using an anti-myeloperoxidase antibody (Abcam ab25989) and an anti-histone H3 (citrulline2 + 8 + 17) antibody (Abcam ab5103). A goat anti-mouse IgG H&L-Alexa Fluor^®^ 647 antibody (Abcam, ab150115) and a goat anti-rabbit IgG H&L-Alexa Fluor^®^ 488 antibody (Abcam, ab150077) were utilized as secondary antibodies. The DNA was already stained with SYTOX Green after permeabilization. The images were captured using a Leica DMi8 inverted microscope.

### Statistics

The data were expressed as the means ± SEMs and analyzed using Student’s *t*-test or one-way ANOVA followed by the Student–Newman–Keuls test. *P* values < 0.05 were considered significant. All the statistical analyses were performed using GraphPad Prism (version 8.01; GraphPad Software, Inc., La Jolla, CA).

## Data Availability

The data that support the findings of this study are available from the corresponding author upon reasonable request.
